# Long read assemblies of geographically dispersed *Plasmodium falciparum* isolates reveal highly structured subtelomeres

**DOI:** 10.12688/wellcomeopenres.14571.1

**Published:** 2018-05-03

**Authors:** Thomas D. Otto, Ulrike Böhme, Mandy Sanders, Adam Reid, Ellen I. Bruske, Craig W. Duffy, Pete C. Bull, Richard D. Pearson, Abdirahman Abdi, Sandra Dimonte, Lindsay B. Stewart, Susana Campino, Mihir Kekre, William L. Hamilton, Antoine Claessens, Sarah K. Volkman, Daouda Ndiaye, Alfred Amambua-Ngwa, Mahamadou Diakite, Rick M. Fairhurst, David J. Conway, Matthias Franck, Chris I. Newbold, Matt Berriman

**Affiliations:** 1 Wellcome Sanger Institute, Hinxton, UK; 2 Centre of Immunobiology, Institute of Infection, Immunity & Inflammation, College of Medical, Veterinary and Life Sciences, University of Glasgow, Glasgow, UK; 3 Institute of Tropical Medicine, University of Tübingen, Tübingen, Germany; 4 London School of Hygiene and Tropical Medicine, London, UK; 5 Department of Pathology, University of Cambridge, Cambridge, UK; 6 Big Data Institute, Li Ka Shing Centre for Health Information and Discovery, Oxford, UK; 7 KEMRI-Wellcome Trust Research Programme, Kilifi, Kenya; 8 Harvard T.H. Chan School of Public Health, Boston, MA, USA; 9 The Broad Institute of MIT and Harvard, Cambridge, MA, USA; 10 Simmons College, Boston, MA, USA; 11 Faculty of Medicine and Pharmacy, Université Cheikh Anta Diop, Dakar, Senegal; 12 Medical Research Council Unit, Fajara, The Gambia; 13 Malaria Research and Training Center, University of Bamako, Bamako, Mali; 14 Laboratory of Malaria and Vector Research, National Institute of Allergy and Infectious Diseases, National Institutes of Health, Rockville, MD, USA; 15 Weatherall Institute of Molecular Medicine, University of Oxford, John Radcliffe Hospital, Oxford, UK

**Keywords:** Plasmodium falciparum, Long read Assembly, complete genomes, definition of core genome

## Abstract

**Background**: Although thousands of clinical isolates of *Plasmodium falciparum* are being sequenced and analysed by short read technology, the data do not resolve the highly variable subtelomeric regions of the genomes that contain polymorphic gene families involved in immune evasion and pathogenesis. There is also no current standard definition of the boundaries of these variable subtelomeric regions.

**Methods**: Using long-read sequence data (Pacific Biosciences SMRT technology), we assembled and annotated the genomes of 15 *P. falciparum* isolates, ten of which are newly cultured clinical isolates. We performed comparative analysis of the entire genome with particular emphasis on the subtelomeric regions and the internal *var* genes clusters.

**Results**: The nearly complete sequence of these 15 isolates has enabled us to define a highly conserved core genome, to delineate the boundaries of the subtelomeric regions, and to compare these across isolates. We found highly structured variable regions in the genome. Some exported gene families purportedly involved in release of merozoites show copy number variation. As an example of ongoing genome evolution, we found a novel CLAG gene in six isolates.  We also found a novel gene that was relatively enriched in the South East Asian isolates compared to those from Africa.

**Conclusions**: These 15 manually curated new reference genome sequences with their nearly complete subtelomeric regions and fully assembled genes are an important new resource for the malaria research community. We report the overall conserved structure and pattern of important gene families and the more clearly defined subtelomeric regions.

## Introduction

*Plasmodium falciparum* is the most virulent of malaria parasites that infect humans and is responsible for hundreds of thousands of deaths each year ^1^. In 2002, the first reference genome for *P. falciparum* was published using a lab adapted clone (3D7) that originated from an infection in Africa ^2^. More than 2000 published studies have cited this single reference and cover a range of topics from functional and comparative genomics to population structure and drug resistance. Genome variation has predominantly been studied by aligning sequencing reads from strains or isolates to the genome sequence of the 3D7 clone. However, the limitations of using this single clone as a reference to map genome variation from all clinical isolates are largely unknown.

This work is part of the ongoing PF3K project that aims to provide the research community with a comprehensive analysis of whole genome short read sequences from 3000 *P. falciparum* isolates from around the world. Because of the highly polymorphic nature of the subtelomeric and internal *var* gene sequences, most short reads from these areas cannot be aligned to the reference genome.

In this study, we used Pacific Bioscience’s SMRT sequencing technology to generate full genome sequence for 15 isolates comprising 5 long-maintained laboratory isolates and 10 recently cultured clinical isolates from different regions of the world. The long-read technology enabled the assembly of subtelomeric regions that contain highly variable genes involved in immune evasion and pathogenesis. We define a core region for each chromosome as the part that contains positionally conserved genes and is bounded by subtelomeres that cannot be aligned between isolates. The newly defined subtelomeres exclude several multigene families that were previously thought to be subtelomeric but appear to be in the much less recombinogenic core.

## Methods

### Ethical approval

For all samples written informed consent was obtained directly from adult subjects and from parents or other legal guardians of all participating children, and additional assent was received from children themselves if they were 10 years of age or older. Collection and analysis of the clinical samples from the Guinea (GN01) was approved under 17/CNERS/12 (Comite National d’Ethique pour la Recherche en Sante, Republique de Guinee) and 6462 (Ethics Committee, London School of Hygiene and Tropical Medicine), and for Mali (ML01) under 2013/55/CE/FMPOS (Comite d’Ethique, University of Bamako) and 6462 (Ethics Committee, London School of Hygiene and Tropical Medicine). For the Senegal samples (SN01) the protocol was reviewed and approved by the ethical committees of the Senegal Ministry of Health (Senegal) and the Harvard T. H. Chan School of Public Health (CR-16330-05). The two Cambodian samples (KH01, KH02) are part of a study registered with ClinicalTrials.gov, number NCT00341003. The use of the Kenya parasite isolate (KE01) was approved under SSC 1131 that was reviewed and approved by the KEMRI Ethics Review Committee and University of Oxford Tropical Research Ethics Committee. The Gabon (GA01) sample was approved by the local ethics committee in Lambaréné, Gabon: CERMEL (Centre de Recherche Médicale de Lambaréné), Lambaréné, Gabon ^3^. The isolates Sudan (SD01), Togo (TG01) and Congo (CD01) were culture adapted from diagnostic specimens submitted for routine malaria diagnosis at the University of Tübingen and therefore did not require separate ethical approval. The isolates 3D7, IT, Dd2, HB3, Gb4 and 7G8 are established laboratory strains, and do not require ethical approval.

### Sample preparation and sequencing

All cultured parasite lines were maintained under standard conditions ^4^. 3D7 was from the same stock as the one used for the genome project published 2002 ^2^. IT, Dd2, HB3, GB4, 7G8 are routinely used laboratory isolates ^5– 7^. Clones of KH01 and KH02, previously named KH1-01 and KH2-0, were also used ^8^. Isolate GA01 from Gabon refers to the MOA D2 Clone that was generated by limiting dilution from the MOA bulk cultures ^3^. The isolates from Sudan (SD01), Togo (TG01) and Congo (CD01) were culture-adapted from diagnostic specimens submitted for routine malaria diagnosis to the laboratory of the outpatient clinic of the Institute of Tropical Medicine in Tübingen, Germany. The clinical samples ML01 from Nioro du Sahel, Mali (Lat 15.183, Long -9.550), and GN01 from Faranah, Guinea (Lat 10.040, Long -10.743) were directly obtained from patients presenting at local health facilities. The Senegalese sample SenT021.09 was collected in Thies, Senegal in 2009. The isolate PfKE01, also known as 9106, based on the naming system adopted at KEMR-Wellcome Trust laboratories for clinical *P. falciparum* isolates, was used in previous studies ^9^.

For each sample, we obtained between 6 – 20 ug of high molecular weight DNA and used Illumina and Pacific Biosciences sequencing platforms. For Illumina sequencing we used the PCR-free method ^10^ to generate the libraries, using 0.5 ug of DNA for each. Libraries were sequenced using the MiSeq Illumina platform with 250 bp paired end reads and a fragment length of 500 bp. In general, 3–4 samples were indexed and pooled per run.

For the Pacific Biosciences SMRT technology we selected an ~8 kb fraction for each isolate and sequenced it using P6 polymerase and version 4 chemistry (P6/C4). For most isolates, 5 SMRT cells were used, resulting in over 100x coverage of the genome. DNA samples from *P. falciparum* 3D7 and IT clones were processed with earlier technology (P5/C3 chemistry) using 11 and 12 SMRT cells, respectively.

### Assembly

The raw reads from Pacific Bioscience sequencing were assembled using HGAP ^11^ version 2.3, using default parameters and a genome size of 23.5 Mb. The following steps were performed within HGAP: read correction, assembly with the Celera assembler and further corrections with Quiver.

Post HGAP, the assembly was further improved. First, contigs smaller than 5 kb were excluded. MegaBLAST ^12^ version 2.2.25 was used (with parameters -W 40 –F F, ignoring hits shorter than 400 bp or less than 98% identity) to determine overlaps between contigs. Those that overlapped by more than 90% of their length and 99% identity with another contig were deleted. Overlapping contigs were merged if the overlap region was larger than 2 kb, shared greater than 99% sequence identity and the coverage of the Illumina reads was around 50% of the median coverage value determined across the whole assembly. After resolving overlaps, contigs were ordered using ABACAS2 ^13^ (parameter 1 kb hits, >98% identity, version 2) against the Pf3D7 reference version 3 (from GeneDB ^14^), excluding the sequences where no one-to-one orthologues to other *Plasmodium* species exist. The apicoplast and the mitochondria were circularized with Circlator ^15^ (default settings, version 0.12.0). Single-base discrepancies and insertions or deletions (indels) up to 3 bp were corrected with iCORN2 ^16^ version 0.95, with three iterations using the 250 bp Illumina reads. All of the steps after the HGAP assembly were performed using a bash script (IPA v1; https://github.com/ThomasDOtto/IPA). The Sudan sample (PfSD01) was also assembled with Canu ^17^ (version 1.6; parameters: genomeSize 24m errorRate 0.15).

### Removal of contamination

To eliminate possible contamination, the GC content for each contig not aligned to a reference chromosome was calculated. Next the number of hits to *P. falciparum* genes was counted on each contig. If a contig had no hits to a gene, a megaBLAST search (parameter: -F F E-value 1e-10, version 2.2.25) against the Pf3D7 reference and the human genome was performed. If a contig had no hits against *P. falciparum* genes or the human genome, it was BLAST-searched against the non-redundant database at NCBI and excluded if its top Blast hit was to another organism.

### Annotation

The contig set was annotated with Companion ^18^, June 2016 version with default parameters. Companion used the Pf3D7 reference version from June 2015. Finally manual curation using Artemis ^19^, version 16.0.9, was carried out to correct the over prediction of coding sequences, to manually curate *var* genes, to insert missing genes such as *EBA-165*, to rename some gene products and to correct some gene borders.

### Bioinformatics analysis

To identify potentially new genes and to enumerate the numbers of genes in gene families, the conceptual proteomes of the 15 isolates together with that of Pf3D7 were clustered using OrthoMCL ^20^ (version 1.4). ** The amino acid sequences were compared using BLASTp all-against-all, version 2.2.25, with an E-value cut-off of 1e-6. To obtain the one-to-one orthologues to *P. vivax* P01 ^21^, the same parameters were used.

Comparative analysis was performed using the Artemis Comparison Tool (ACT) ^22^, (version 16.0.9), by comparing the new assemblies to each other and to the Pf3D7 reference.

Multiple alignments were generated using Mafft ^23^ (version 7.205, parameter --auto) and trimmed with GBLOCKS ^24^ (version 0.91b) in Seaview ^25^ (version 4.6.1) before building phylogenetic trees with RaxML ^26^ (version 8.0.24) and 100 bootstrap samples. RaxML was also used to estimate the best fitting model. Visualization was done in FigTree ^27^ (version 1.4.2).

Networks of BLASTp hits (version 2.2.25, with an E-value cut-off of 1e-6) were clustered with the Fruchterman algorithm option and visualized with Gephi ^28^ (version 0.9.1).

To compute the global distribution of a novel gene that was absent in Pf3D7, we used the Pf3K dataset ( ftp://ngs.sanger.ac.uk/production/pf3k/release_5/). All non-mapping reads were extracted with samtools view -f 12 (version 0.1.19-44428cd) from the downloaded bam files. The reads were mapped with BWA-MEM ^29^ (version 0.7.12-r1039) against the nucleotide sequence of PfIT_110029200 (one allele of the novel gene). If 10 reads from a Pf3K sample mapped to that gene it was assumed to be present. The novel gene was further analysed using Pfam ^30^ and I-Tasser ^31^ using the webpages from December 2017, with default settings.

### Assembly evaluation and SNP calling

We evaluated the assembly quality by comparing our novel long read assemblies of Pf3D7 with that of the reference genome. ****Regions of large-scale miss-assembly were identified by parsing the OrthoMCL output and visually inspecting the regions in ACT. To detect 1–5 base pair errors, we used the GATK pipeline ^32^, version v3.4-46-gbc02625, following the best practice of GATK. We were not able to perform the SNP calling on alignments of complete chromosomes against Pf3D7 because of the extended runtime required (>1 day). We therefore called variants by cutting the genomes into 50kb sliding windows with a 1kb offset, generating 8x coverage on each strand (16x in total). As the assembled contigs/chromosomes did not have quality values, we used a default base quality score of 30. The resulting variant call format (VCF) file was used to find differences between the Pf3D7 reference and the PacBio assemblies, either before or after correction with iCORN2.

### Definition of the core genome

To define core conserved regions for each chromosome, the consensus sequences for each isolate were mapped with BWA-MEM (as above) against the corresponding Pf3D7 chromosome. The outer limits of the core for each chromosome were defined as the most telomere-proximal position covered by the majority (8 out of 15) of these aligned sequences. Regions of the subtelomeres that bound to heterochromatin protein 1 were obtained from the GEO repository (accession: GSE102695 ^33^).

To generate the plots of Figure 3B, the above BWA-MEM alignments were used. The leftmost or rightmost position when a mapping was clipped (soft or hard) was used as the point at which synteny was lost. Where there was no clipping at the end of an alignment, (the assembly did not reach into the subtelomere), that isolate at that chromosome end was excluded. The cumulative distribution of mapping termination sites was then plotted using R (version 3.1.2).

## Results

### Evaluation of assembly approach

**Figure 1.**
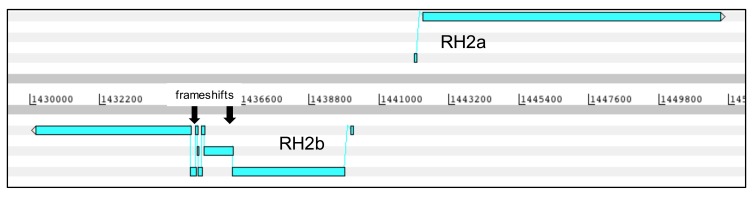
Frameshifts in the *Rh2b* gene that could not be corrected in the Pf3D7 PacBio assembly. Most of the *Rh2b* sequence is identical to *Rh2a.* Read correction approaches like quiver or iCORN2 fail resulting in seven apparent frameshifts (arrows).

The genome reference clone 3D7 was resequenced to assess the performance of using a combination of long (> 5kb, median 8kb) and short-read sequencing technologies to produce complete *P. falciparum* genome assemblies. The 14 nuclear chromosomes and 2 plastid genomes were represented in just 23 contigs. The contigs were aligned against the reference genome using BWA-MEM and variants called with GATK. Although ~25,000 indel discrepancies and ~1800 single nucleotide substitutions ( Table 1) were initially found, correcting the contigs by iteratively mapping Illumina reads reduced the number of indels to ~5,800 and the single-nucleotide substitutions to ~1,000. The error rate in PacBio sequencing is known to increase in homopolymer regions (long runs of a single base), and such features (more than 6 identical nucleotides in a row) accounted for 93% of the indel discrepancies. Since 98% of homopolymer errors were in homopolymer tracks of >14bp these sequences were excluded from subsequent analyses. In addition, we observed large numbers of errors in TA repeat sequences. 96% of these errors were in repeats > 14bp, so they were also excluded from the analysis. Homopolymers and (TA) _7+_ repeats together comprised 5.6% of the reference genome sequence. Following this correction and filtering, approximately 1100 single nucleotide or indel discrepancies remained between the 3D7 PacBio assembly and the 3D7 reference genome ( Table 1). A small number of larger errors were seen: a misassembled region within the repetitive rRNA gene cluster on chromosome 11 that could be bridged by longer reads in subsequent sequencing runs; a deletion at the end of chromosome 9 – a region that has previously been lost during *in vitro* growth ^34^; and frameshift errors in duplicated regions, notably the Rh2a and Rh2b genes that have largely identical sequences ( Figure 1). Differences in the length of the apicoplast sequences were also observed ( Table 2). We anticipate that the correct size is around 34 kb, but our pipeline was unable to resolve the assembly of an inverted repeat > 5kb. No other sequences were found to be missing and, as detailed below, several large-scale differences between the PacBio assembly and the Pf3D7 reference were subsequently attributed to errors in the reference genome.

**Table 1.** Comparison of raw and iCORN2 corrected assemblies against the 3D7 reference genome. Using GATK, variants were called from the PacBio assembled contigs of 3D7 aligned against the 3D7 reference genome with and without TA-repeats and homopolymer tracks excluded. After iCORN2 correction the vast majority of variant sites were no longer present demonstrating the high accuracy of the PacBio + iCORN2 assembly approach.

	GATK calls using all bases		GATK calls (excluding long homopolymers and TA repeats)	
	Pre iCORN2	Post iCORN2	Pre iCORN2	Post iCORN2
Single-base differences	1,798	1,020	945	697
Indels	25,352	5,857	11,093	401

**Table 2.** Overview of assemblies from 15 *Plasmodium falciparum* isolates.

Isolate	Synonyms	Total assembled bases *	Contigs	Chromosomes with 2 telomeres attached	Centromeres	Protein- coding genes	Apicoplast assembly length	Mitochondrion assembly length	VAR genes (> 2.5 kb) **
Pf3D7 (v3.1)		23,292,622	15	14	13	5,432	34,247	not present	67
Pf3D7 (Pacbio)		23,199,454	23	6	14	na	34,249	6,093	na
Guinea (GN01)		23,672,855	19	14	14	5,455	34,242	6,094	90
Congo (CD01)		23,545,947	19	11	14	5,397	not present	not present	75
Dd2	Dd2-2D4	22,641,838	16	10	14	5,297	34,247	6,094	51
Kenya (KE01)	9106	22,736,618	25	11	14	5,312	45,629	6,087	57
Cambodia (KH01)	KH1-01	23,225,979	22	6	14	5,417	34,248	6,094	68
7G8		22,774,710	17	9	14	5,318	51,008	6,094	47
Gabon (GA01)	MOA D2	23,089,490	20	8	14	5,362	51,533	6,094	64
GB4		23,436,708	26	6	14	5,447	46,656	6,094	74
Cambodia (KH02)	KH2-01	23,068,186	21	10	14	5,367	50,279	6,095	56
IT		23,123,850	27	5	14	5,327	51,087	6,094	60
Sudan (SD01)		22,709,431	19	5	14	5,292	34,244	6,094	56
Togo (TG01)		25,900,321	79	5	14	5,944	34,245	6,094	147
HB3		22,771,623	28	3	14	5,299	34,247	6,094	50
Senegal (SN01)	SenT021.09	23,450,349	36	4	11	5,432	43,092	6,093	79
Mali (ML01)		25,622,360	117	0	14	5,882	46,599	6,094	130

* Total bases exclude apicoplast and mitochondrion.

** VAR genes (>2.5kb) includes all *var* sequences above stated length (i.e. intact genes, pseudogenes and fragments).

### Genome assemblies from 15 unrelated isolates

High quality genome assemblies were produced from 15 globally sampled *P. falciparum* isolates – ten clinical isolates recently adapted to *in vitro* cultivation and five commonly used laboratory clones ( Table 2). The genomes of 13 isolates assembled into just 16–36 contigs (median = 21). Two isolates (PfTG01 and PfML01) comprised multiple infections and were excluded from the analysis unless stated. In the genomes assembled from single infections 5,300 – 5,700 genes were found without any spanning sequencing gaps. For 4,700 genes, clear one-to-one orthologues could be found using OrthoMCL, reflecting the high quality of the assembly and annotation. Many of the contigs terminated with telomeric repeats at either end and therefore appear to represent complete chromosomes ( Table 2). Some chromosomes (e.g. chromosome 12) consistently appear to be complete in the assemblies across several isolates, whereas chromosome 6 was particularly problematic to assemble ( Table 3).

Within the 15 new genome assemblies we found two that had an error in the rRNA gene on chromosome 11, as we had observed in our assembly of Pf3D7. Using reads produced with the newer C4P6 PacBio chemistry that had a longer median length (≥ 12kb), the region was correctly assembled. Another large-scale misassembled region between chromosome four and seven, resulted from using HGAP to assemble PfSD01. Two contigs that shared a 12-kb region that is duplicated in the genome were incorrectly merged. With the use of an alternative assembler (Canu, see methods) the region was resolved into two contigs.

**Table 3.** Summary of assembly-completeness for 13 *P. falciparum* lines assembled *de novo* from PacBio data. All isolates included except ML01 and TG01, which appear to be mixed infections. Left and right telomeres are arbitrarily defined based on the 3D7 reference that was used throughout.

Chromosomes	Completely assembled	Left telomere present	Right telomere present
1	6	9	7
2	6	8	8
3	7	10	8
4	7	10	9
5	5	7	9
6	4	7	7
7	8	12	8
8	6	6	9
9	7	8	10
10	6	9	9
11	8	11	9
12	10	12	11
13	6	9	9
14	10	13	10

**Figure 2.**
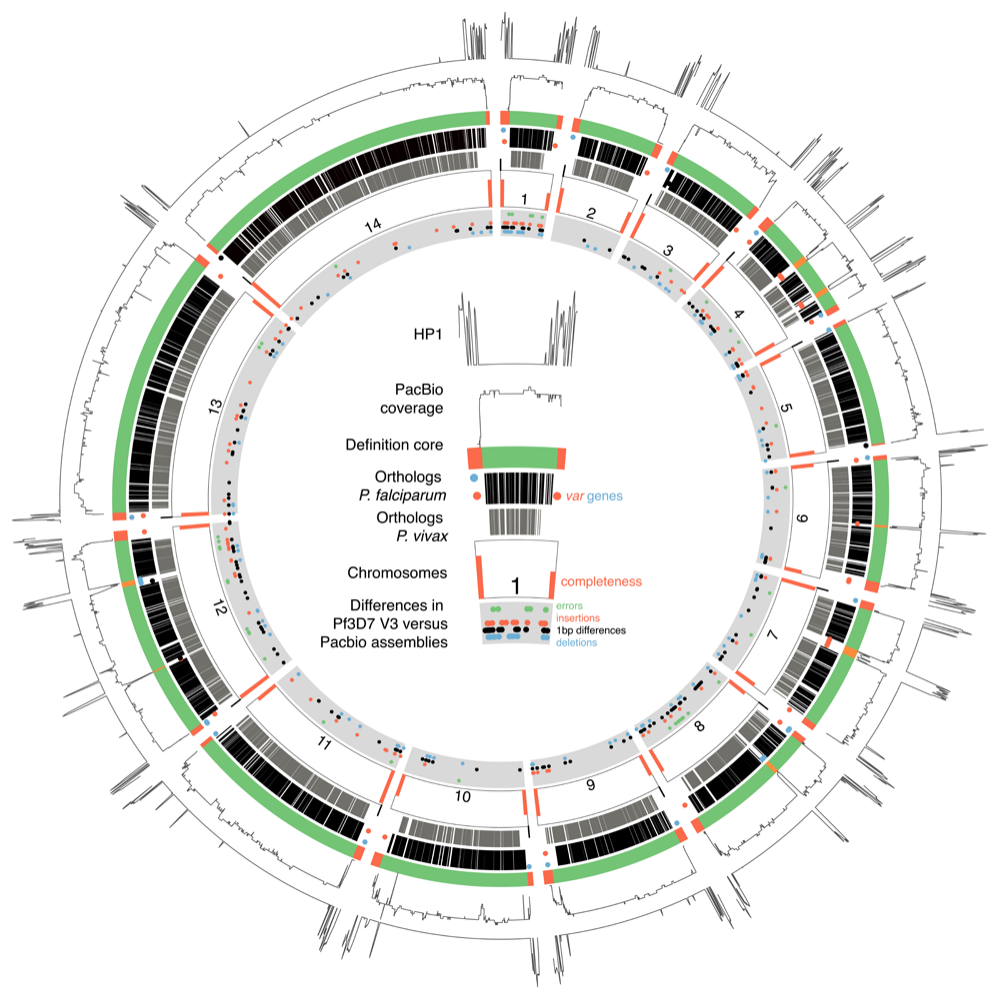
Summary for evidence for *Plasmodium* core definition. From outside to inside the figure represents: heterochromatin protein 1 binding sites in Pf3D7; the coverage of mapped reads from PacBio genomes used to define the core region; our new definition of core regions (green) and the subtelomeres or internal *var* gene cluster (orange); one-to-one orthologues with *P. falciparum* (black lines) or *P. vivax* (grey lines). The dots in this latter track are *var* genes (≥2.5kb) coloured blue if on the forward strand, red if on the reverse strand, and black if a pseudo gene. Chromosome numbers are shown together with orange bars –the height of which indicates the proportion of subtelomeres in the PacBio assemblies that were complete. The innermost track shows differences between the 3D7 reference genome and the PacBio assemblies: green depicts sites where all isolates are different to the reference, orange shows insertions, black shows 1 bp differences and blue depicts insertions after masking homopolymer tracks and TA repeats >14bp.

Comparing all of the PacBio based assemblies to the Pf3D7 reference, 104 single nucleotide and indel discrepancies were consistently found indicating that these are likely to be errors in the current reference genome ( Figure 2).

**Figure 3.**
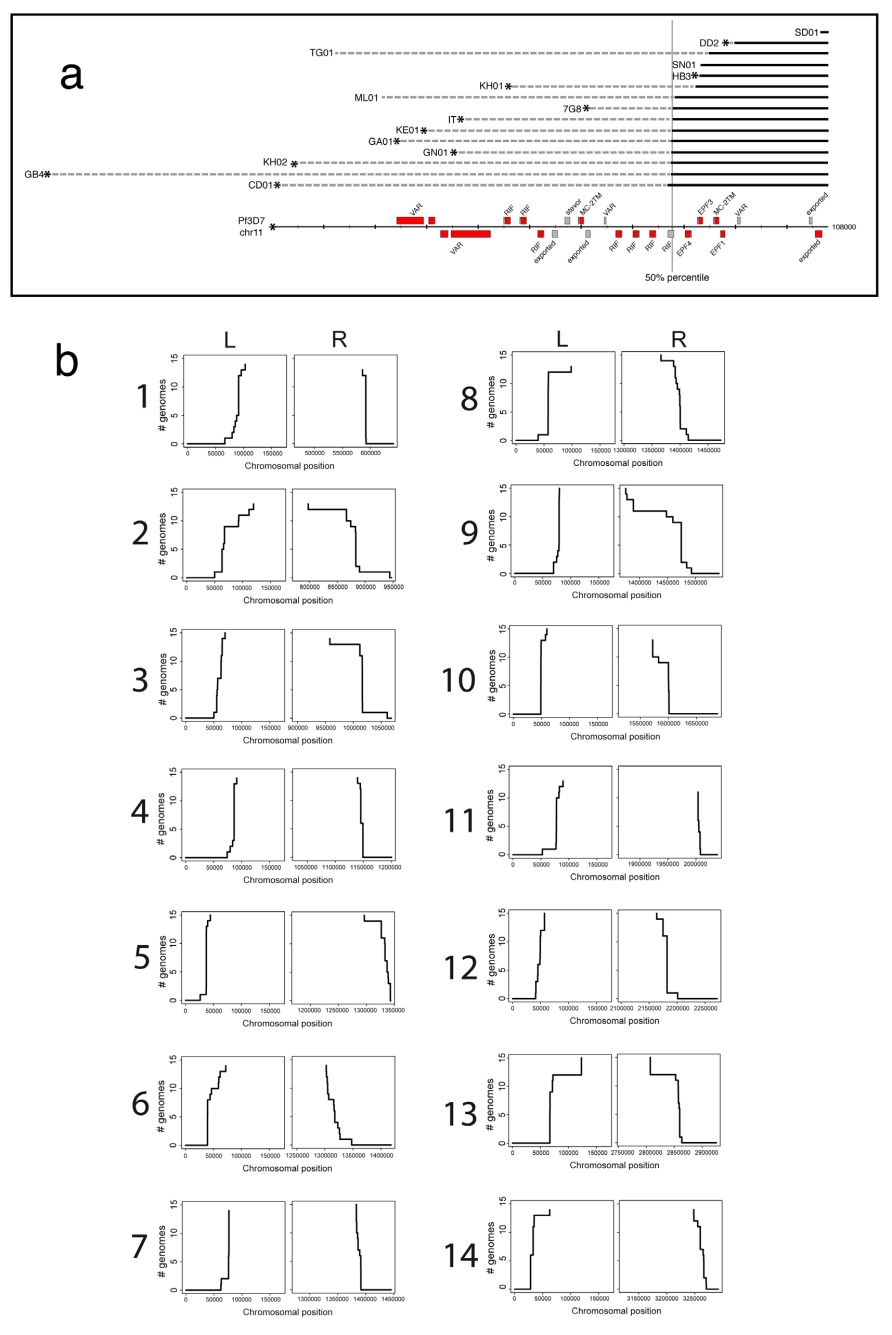
Definition of the core genome. ( **A**) 15 assemblies (GN01, CD01, DD2, KE01, KH01, KH02, 7G8, GA01, GB4, IT, SD01, HB3, SN01, ML01, TG01) mapped to the left hand side of chromosome 11 (chr11) of *P. falciparum* 3D7. A general core boundary is being defined as the point closest to a telomere at which 8/15 genotypes cease to align (50% percentile). ( **B**) Cumulative distributions of termination positions of mapping between each isolate and the 3D7 reference are shown for each chromosome end (L = left, R = right). Where mapping terminated without clipping the isolate was excluded.

### Chromosome structure

Genes within *Plasmodium* species can be subdivided into two broad (but not exclusive) categories: genes with one-to-one orthologues across the genus that are generally positionally conserved and genes that have expanded either across the genus (e.g., the *pir* genes that include *rif* and *stevor* in *P. falciparum*), or in specific clades or species (e.g. *var* in *P. falciparum*). Expanded gene families are typically highly polymorphic and located within the subtelomeres or in internal clusters.

**Figure 4.**
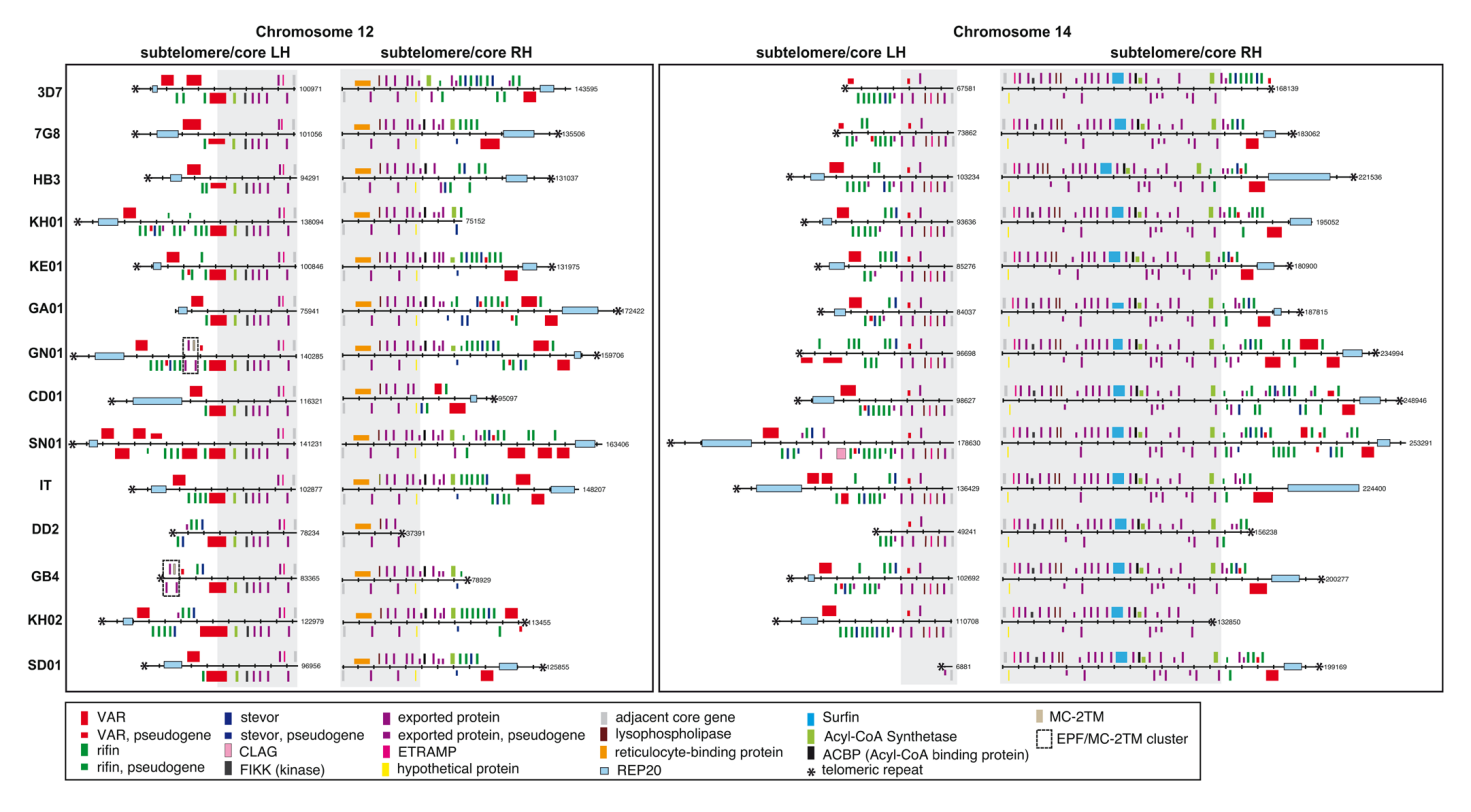
Example of core/subtelomere definitions for chromosomes 12 and 14. The grey areas represent the boundaries of newly defined core regions. The order and orientation of genes and repeats on the left- and right-hand sides of chromosomes 12 and 14 are shown in *P. falciparum* 3D7 *,* 7G8, HB3, KH01 (Cambodia), KE01 (Kenya), GA01 (Gabon), GN01 (Guinea), CD01 (Congo), SN01 (Senegal), IT, Dd2, GB4, KH02 (Cambodia), and SD01 (Sudan) are shown. Genes are represented as coloured boxes. The subtelomere/core areas either end or start with the first gene, with orthologs in more than one *Plasmodium* species outside of *P. falciparum.* These are the following gene IDs in *P. falciparum* 3D7: PF3D7_1201500, PF3D7_1252300, PF3D7_1401700, PF3D7_1475900.

To define the latter regions, we used the lack of ability to align chromosome assemblies from long PacBio reads of different genotypes to 3D7. Consensus sequences were aligned to the 3D7 reference and used to identify region boundaries for each chromosome as the point at which the assemblies cease to align. In general, these points are remarkably close together or identical regardless of the genotype being aligned (for complete data see Figure 3A and B) but for simplicity we hereafter define a general boundary as the point closest to a telomere at which 8/15 genotypes cease to align. Together with a detailed manual examination this analysis clearly partitions the genome into three distinct parts with defined boundaries; long regions that are conserved and co-linear between isolates (hereafter called the core), internal clusters of *var* genes, and the subtelomeres ( Figure 2, Table 4). It therefore appears that the core region of conserved genes for each chromosome extends much further towards the telomeres than previously thought ^7^ and that an abrupt increase in recombination (indicated by contigs for each isolate extending but no longer aligning) defines the true start of the subtelomere. This definition of the subtelomeres closely corresponds with the region known to bind heterochromatin protein 1, a protein whose location is limited to those areas harbouring genes that show mutually exclusive or highly heterogeneous expression patterns ^33^ ( Figure 2). Figure 4 illustrates the subtelomeric boundary in more detail at the ends of chromosomes 12 and 14. Several multigene families, such as FIKK, SERA, Acyl-CoA synthase and SURFIN that have formerly been regarded as subtelomeric, do not appear to be in the highly recombinogenic part of the genome.

**Table 4.** Coordinates of the core region for each 3D7 chromosome. The internal *var* gene clusters are not part of the core.

Chromosomes	Core from .. to	Internal *var* gene cluster	
		First	Second
Pf3D7_01_v3	91183..591736		
Pf3D7_02_v3	67438..880726		
Pf3D7_03_v3	63099..1016045		
Pf3D7_04_v3	86540..1145399	546226..601217	936358..979881
Pf3D7_05_v3	37149..1333478		
Pf3D7_06_v3	39483..1317743	724169..742098	
Pf3D7_07_v3	76561..1386108	512373..591896	
Pf3D7_08_v3	57343..1391320	430803..466095	
Pf3D7_09_v3	79027..1448081		
Pf3D7_10_v3	49338..1600156		
Pf3D7_11_v3	77654..2003815		
Pf3D7_12_v3	49282..2175030	768014..775380	1695106..1741581
Pf3D7_13_v3	72312..2857354		
Pf3D7_14_v3	32983..3259777		

**Figure 5.**
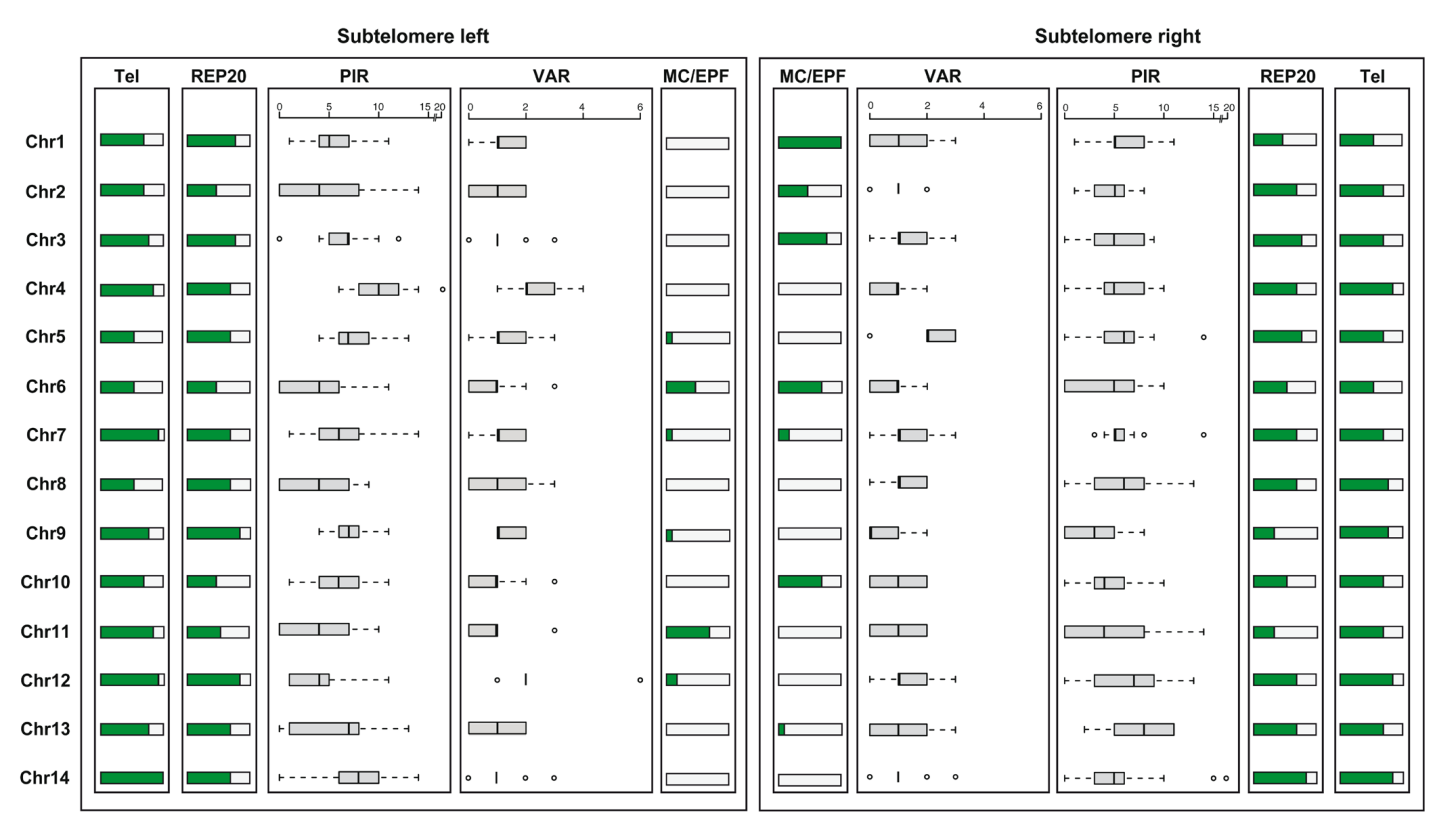
Overview of subtelomeres in new assemblies. The figure shows the left and right subtelomere of 13 assemblies (GN01, CD01, DD2, KE01, KH01, KH02, 7G8, GA01, GB4, IT, SD01, HB3, SN01). The proportion of chromosomes with telomeric repeats (Tel) and REP20 repeats (REP20), the distribution of the number of PIR- and *var*-like sequences (including both genes and pseudogenes) and the presence of cassettes consisting of EPF1 (exported protein family 1), MC-2TM (MC-2TM Maurer's cleft two transmembrane protein), EPF3/Hyp4 (exported protein family 3) and EPF4/HYP5 (exported protein family 4) are shown (MC/EPF). PIR includes *rif* and *stevor* genes.

**Figure 6.**
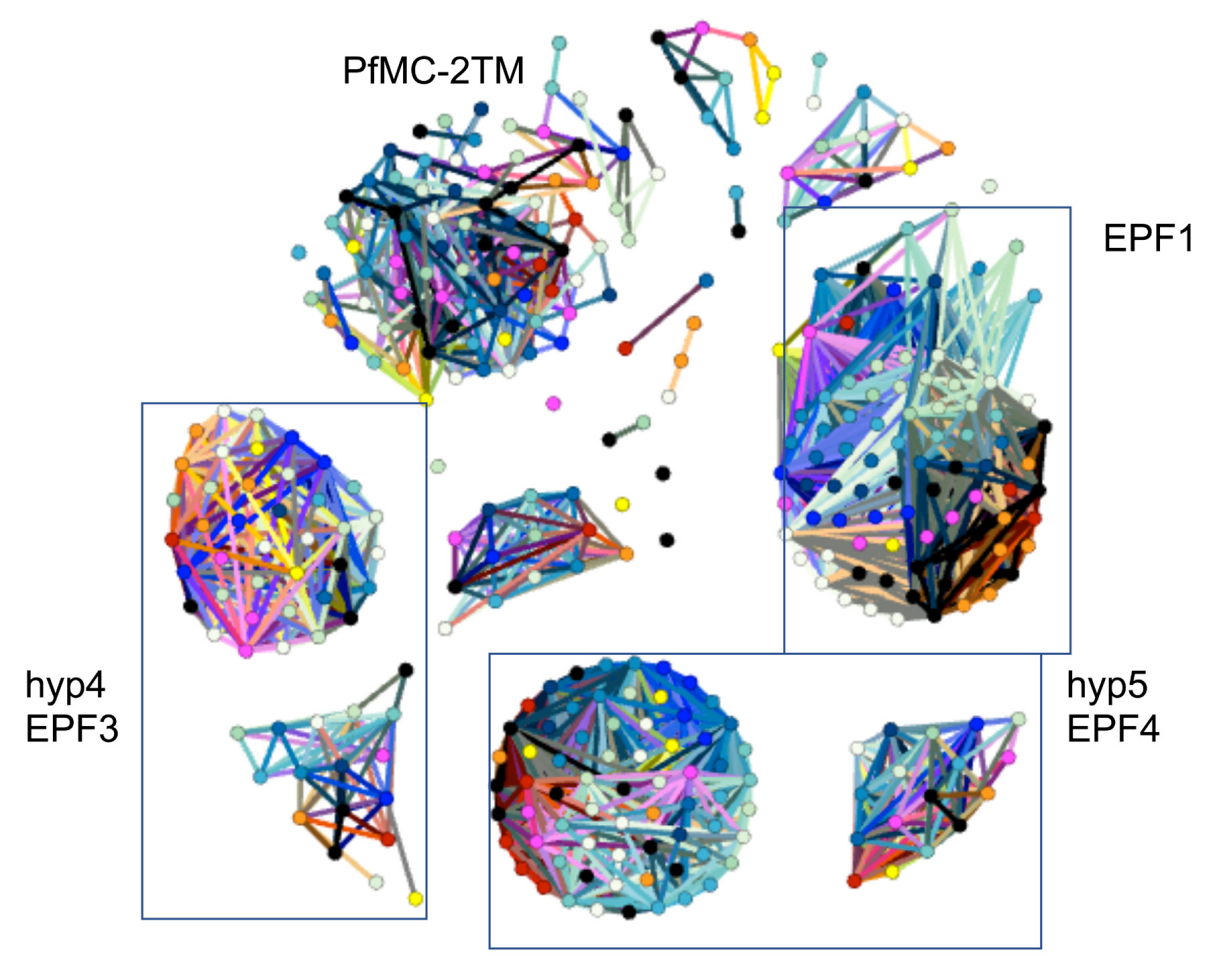
Similarity graph of exported proteins EPF1, EPF3, EPF4 and PfMC-2TM. Each node represents a protein. Proteins are connected where they share ≥ 94% global identity.

The “newly defined” subtelomeres have a similar structure but variable length across the isolates; telomeric heptamer repeats are in most cases followed by repetitive regions of several thousands of base pairs including REP20 ^35^ ( Figure 5), followed by one *var* gene that is transcribed towards the centromere ( Figure 4). The numbers of *var, rif* and *stevor* genes in each of the subtelomeres are variable, as are the overall numbers per genome: 47-90 *var* genes ≥ 2.5kb (median 62), 122-185 *rif* genes ** (median 152) and 22-44 *stevor* genes (median 40) ( Table 5). Genes considered subtelomeric by our definition include the PfMC-2TM genes and other families encoding exported proteins ( Table 5). As previously described ^36^, alleles of EPF1 (export protein family 1), PfMC-2TM, EPF3 and EPF4 commonly occur next to each other as a gene cassette. We found that these genes occur as cassettes in all genomes but with the number of cassettes varying from 4 to 9. In 3D7, 3.5 out of 9 of these cassettes are part of the subtelomeres. A network analysis ( Figure 6) revealed the level of sequence identity and sub-clustering that occurs in these genes. PfMC-2TM is the most diverse, and in the similarity graph we observe several small clusters. While the function of this group of four genes is not fully understood, Mbengue and colleagues reported that down-regulation of EPF1 is associated with inefficient release of merozoites ^36^.

**Table 5.** Distribution of genes with multiple copies, based on the current annotation (13 *Plasmodium falciparum* lines).

	3D7				*P. falciparum* isolates												
Multigene family	all	core	sub	VAR array	IT	HB3	DD2	GB4	7G8	GN01	CD01	GA01	SN01	SD01	KH01	KH02	KE01
Rifin (RIF)	184	16	158	10	135	122	136	185	126	185	169	138	176	132	184	162	143
Plasmodium exported protein/ Plasmodium RESA N-terminal	128	110	18	0	80	66	70	75	79	75	70	71	56	66	53	72	65
Erythrocyte membrane protein 1 (PfEMP1)	105	20	62	23	72	76	72	104	68	116	99	94	118	72	93	75	83
Erythrocyte membrane protein 1 (> 2.5 kb)	67	6	38	23	60	50	51	74	47	90	75	64	79	56	68	56	57
Plasmodium exported protein (PHIST)	80	65	15	0	57	62	70	59	59	57	57	53	59	59	58	54	56
Stevor	42	11	30	1	40	31	40	38	22	44	43	40	43	23	41	40	35
EPF1 (exported protein family 1)	13	9	4	0	8	8	6	11	5	11	7	11	10	8	8	8	8
Maurer's cleft two transmembrane protein (MC-2TM)	13	8	5	0	9	9	7	11	6	12	9	12	11	9	9	9	9
EPF4/HYP5 (exported protein family 4)	9	5	4	0	6	5	4	7	4	10	8	7	5	6	6	4	6
EPF1/MC-2TM/ EPF3/EFP4 cluster	9	5.5	3.5	0	6	4	4	7	4	9	7	6	4	5	6	4	6
EPF3/HYP4 (exported protein family 3)	9	5	4	0	7	8	5	8	4	10	8	9	6	7	7	5	6
Plasmodium exported protein (HYP)	33	33	0	0	32	33	34	32	32	32	32	30	32	32	33	32	28
Acyl-CoA synthetase	13	12	1	0	14	10	12	14	13	13	12	12	13	12	14	12	14
Lysophospholipase	6	4	2	0	8	9	10	9	9	9	9	7	9	8	9	9	7
Cytoadherence linked asexual protein (CLAG)	5	5	0	0	6	6	5	4	7	5	5	6	5	5	5	5	3
Surface-associated interspersed protein (Surfin)	10	10	0	0	11	11	10	11	10	11	11	10	11	13	11	11	10
Serine/threonine protein kinase, FIKK family	21	21	0	0	21	19	21	21	21	21	21	21	21	20	21	22	20
Early transcribed membrane protein (ETRAMP)	15	15	0	0	14	14	14	14	14	14	14	13	14	13	14	13	13
Reticulocyte binding protein	7	7	0	0	7	7	7	7	7	7	7	7	7	7	5	7	7
Serine repeat antigen (SERA)	9	9	0	0	9	9	9	9	9	9	9	9	9	9	9	9	9
Erythrocyte binding antigen *	5	5	0	0	5	5	5	5	5	5	5	5	5	5	5	5	5
AP2 domain transcription factor	27	27	0	0	27	27	27	27	27	27	27	27	27	27	27	27	27
MSP7 and MSP7- like protein **	9	9	0	0	9	9	9	9	9	9	9	9	9	9	9	9	9
6-cysteine protein	14	14	0	0	14	14	14	14	14	14	14	14	14	14	14	14	14

* includes EBA140, EBA175, EBA165, EBA181, MAEBL. Identified using Pfam (domain PF11556)

** identified based on Pfam domain (PF12948)

**Figure 7.**
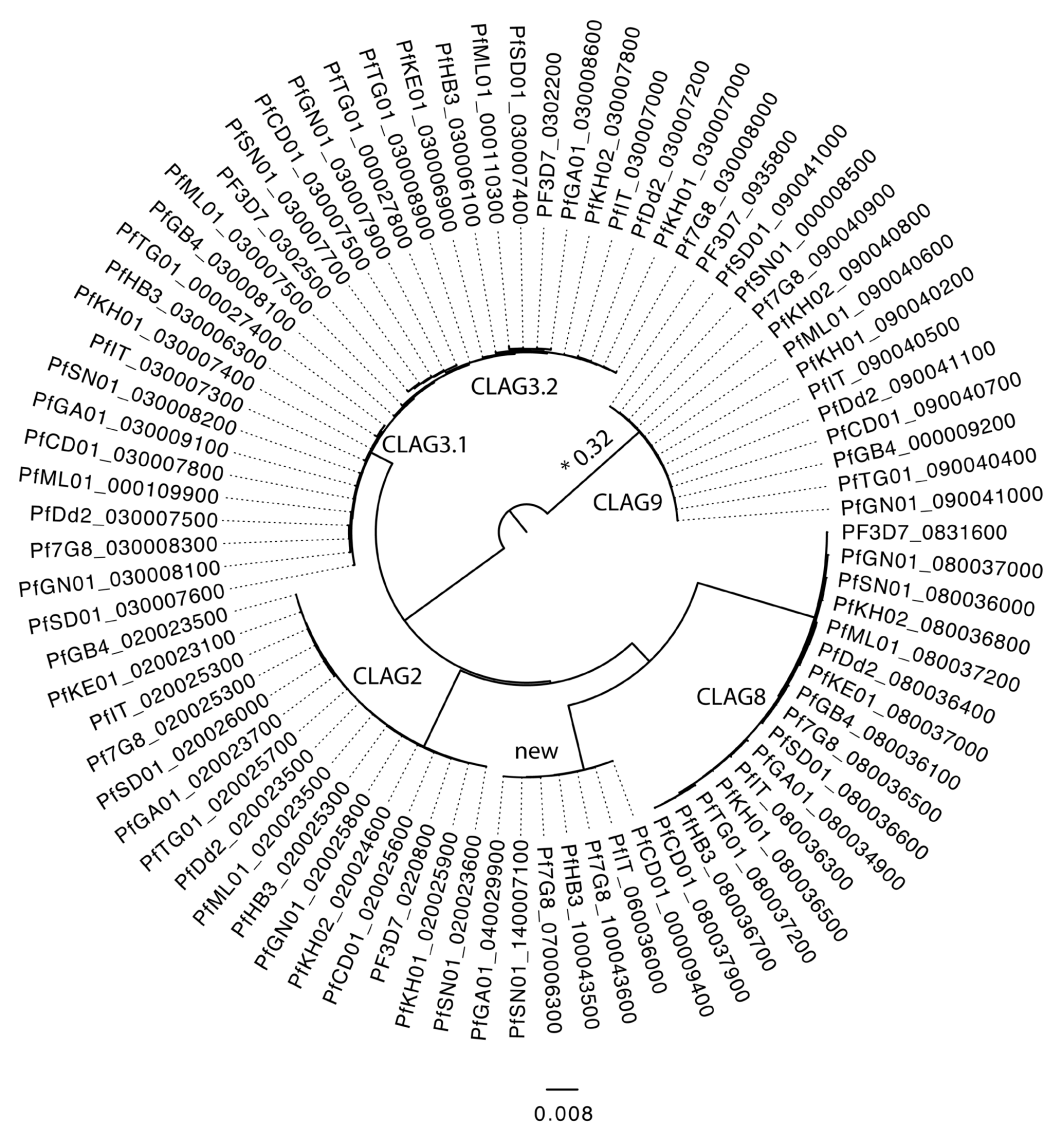
Phylogenetic tree of Cytoadherence Linked Asexual Gene (CLAG) genes including the novel CLAG (new). The new CLAGs are PfDC01_00009400, PfIT_060036000, PfHB3_100043500, Pf7G8_100043600, PfSN01_140007100, PfGA01_040029900 and Pf7G8_070006300. The tree was built with the LG4XF model and all nodes have a bootstraps value of 100. The branch with * was shortened by a factor of 5, from 0.322.

The Cytoadherence Linked Asexual Gene (CLAG) multigene family is found across the *Plasmodium* genus ( Figure 7). Despite their historic name these genes are now known not to be involved in cytoadherence. CLAG9 encodes a protein known to be a component of the rhoptry ^37^ whereas CLAG3 has been reported to be expressed in the infected red cell membrane and to be involved in nutrient uptake ^38^. In addition to five known CLAG genes that are positionally conserved within the core region of *P. falciparum*, we identified a novel CLAG on different subtelomeres in our new assemblies. Given the broad range of important parasitological roles of characterised CLAG family members, determining the function of this new uncharacterised CLAG gene will be of great interest.

**Figure 8.**
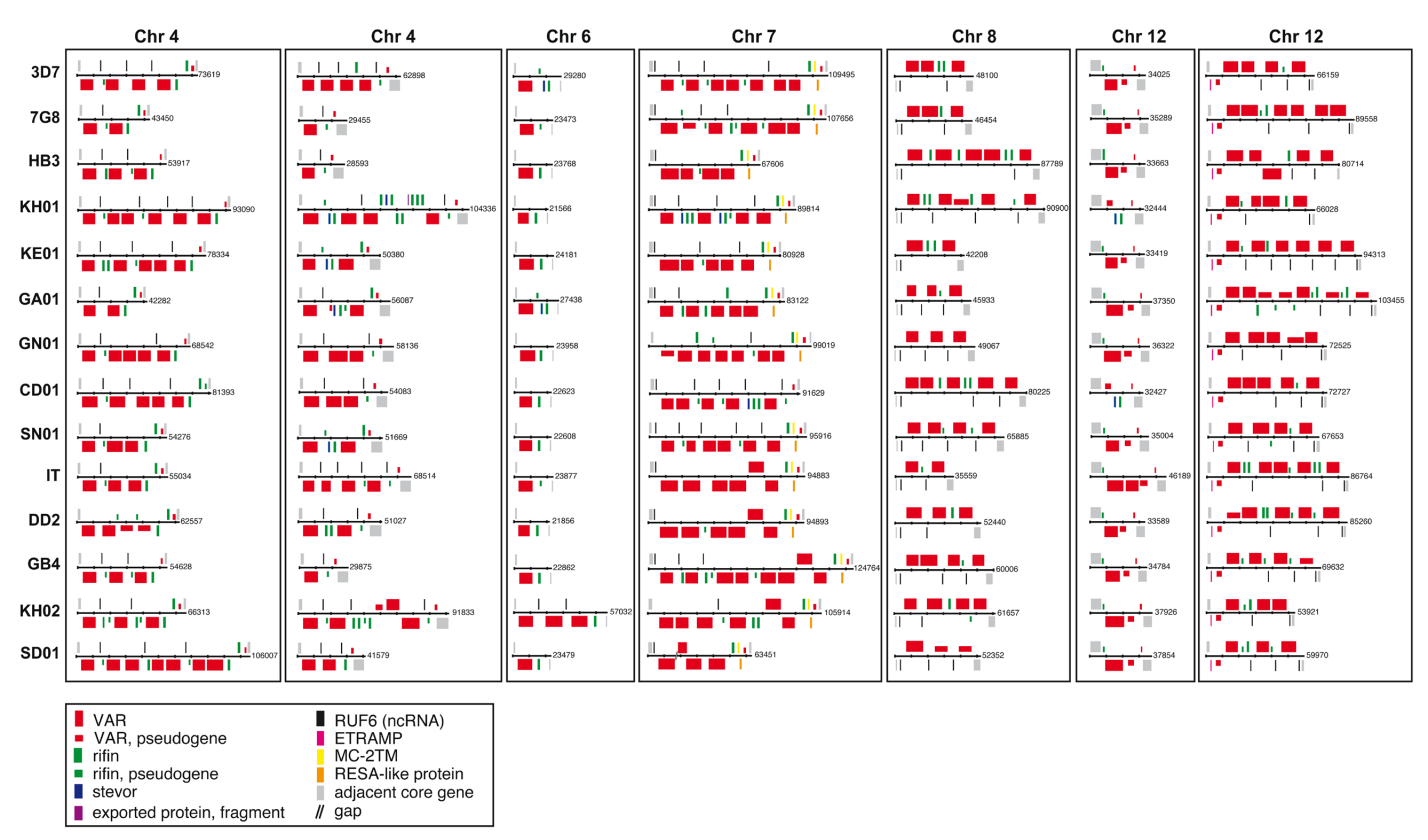
Overview of internal *var* gene arrays in 13 *Plasmodium falciparum* genomes. The order and orientation of genes on 7 internal *var* arrays in *P. falciparum* 3D7, 7G8, HB3, KH01 (Cambodia), KE01 (Kenya), GA01 (Gabon), GN01 (Guinea), CD01 (Congo), SN01 (Senegal), IT, Dd2, GB4, KH02 (Cambodia), and SD01 (Sudan) are shown. Genes are represented as coloured boxes.

Our assemblies also revealed the conserved arrangement of the seven internal *var* gene clusters ( Figure 8) that are clusters with at least one functional *var* gene, within the core regions of the genome. No new internal *var* clusters were found. The orientation of the *var* genes within them were generally conserved with only six exceptions out of more than 300 genes. Four of the seven clusters have a highly conserved *var* pseudogene on the reverse strand that delimit the cluster boundary.

### Differences between isolates within the core

**Figure 9.**
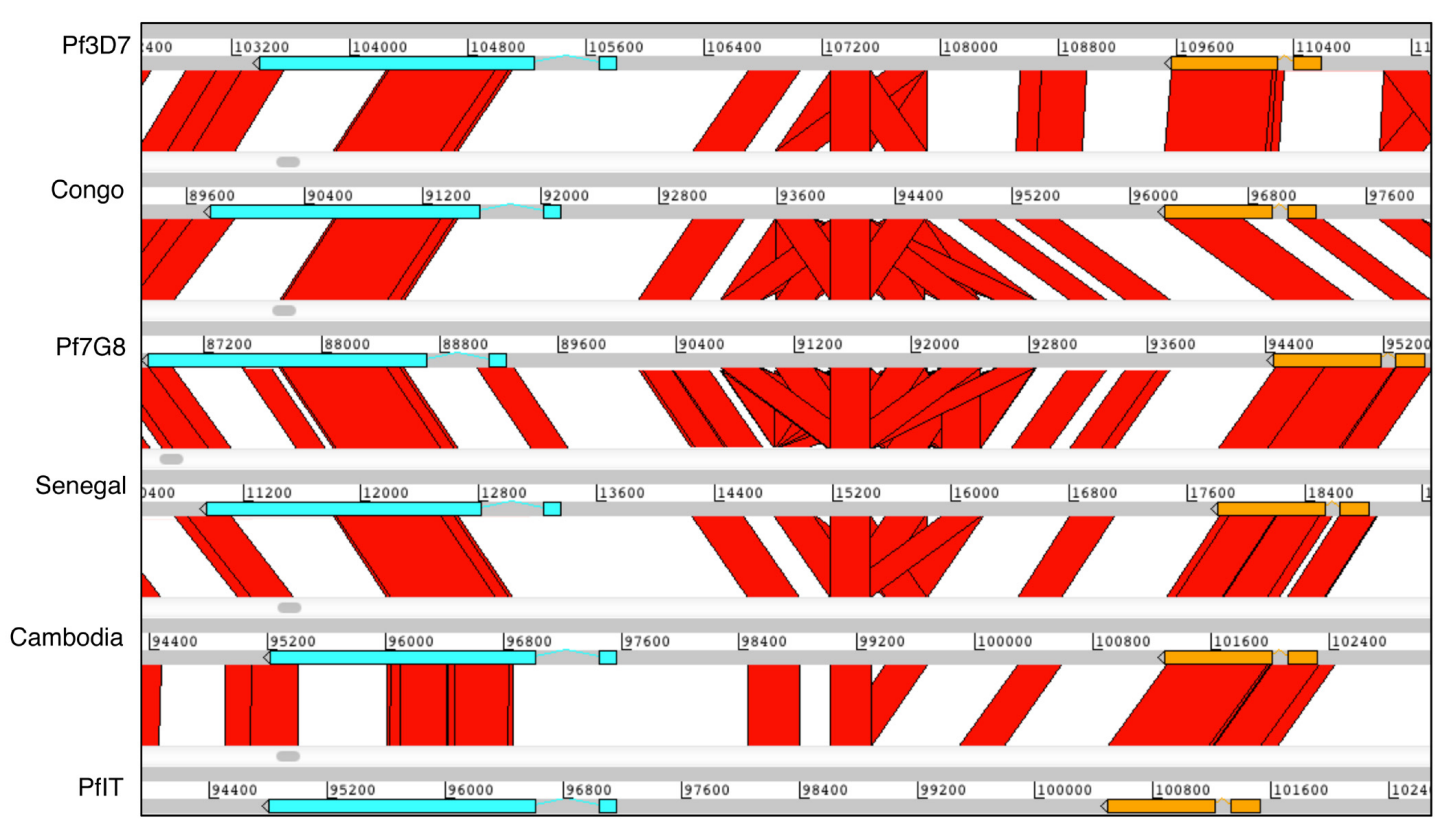
KAHRP promoter copy number variation. Comparisons of *Plasmodium falciparum* 3D7, CD01 (Congo), 7G8, SN01 (Senegal), KH01 (Cambodia), IT shows copy number variation in the KAHRP (knob-associated histidine-rich protein) promoter. ACT (Artemis Comparison Tool) comparison of chromosome 2 shows copy number variation in the KAHRP promoter. Grey bars represent the forward and reverse DNA strands. The red blocks between sequences represent sequence similarity (tBlastx). KAHRP is shown in blue. There is a difference in the number of copies: The number of copies is as follows: 1 copy (IT, DD2, GB4), 2 copies (GN01, KH01, KH02, SD01, HB3, 3D7), 3 copies (CD01, GA01), 4 copies (SN01) and 6 copies (7G8).

We did not find any genomic transpositions in the core. A few large deletions were found, like some reticulocyte binding proteins in KH01 ( Table 5), at the KAHRP (knob-associated histidine-rich protein) locus in isolate Dd2 and near the end of chromosome 9 where several isolates have deleted a segment containing a number of genes as previously reported ^34^. We were unable to find duplications larger than the median read length of 12 kb, since these would have been collapsed into single copies within our assemblies. By manual inspection we found a copy number variation in the promoter region of KAHRP in several isolates ( Figure 9). Whether this observation relates in any way to knob density at the red cell membrane remains to be determined.

Due to the completeness of our assemblies, it was possible to characterize other core genes that have been previously difficult to access. For example, the region on chromosome 10 (position 1,391,000 – 1,463,000) harbours many genes involved in merozoite invasion. With short reads, these genes are too diverse to be aligned well enough to enable confident SNP calling. As this locus is represented completely in the newly assembled isolates, it will enable the community to analyse those genes in more detail.

### A new gene with a geographic signature

**Figure 10.**
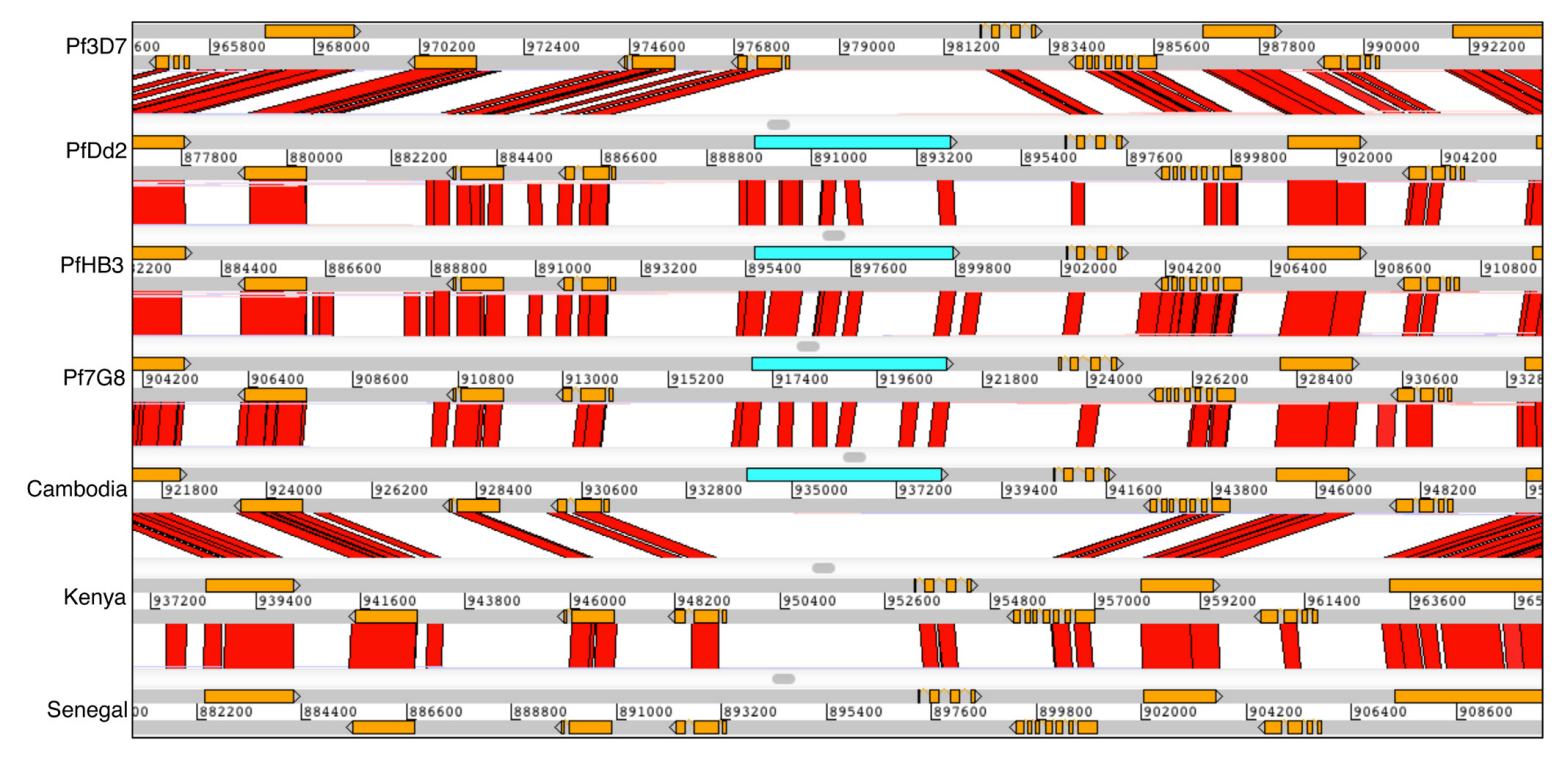
Comparisons of multiple isolates reveal a novel hypothetical gene on chromosome 11. ACT (Artemis Comparison Tool) comparison of chromosome 11 of these isolates shows the position of this gene of unknown function where present (blue). Grey bars represent the forward and reverse DNA strands. The red blocks between sequences represent sequence similarity (tBlastx). The gene is present in Dd2 (PfDd2_110027900), HB3 (PfHB3_110028200), 7G8 (Pf7G8_110028600), KH01 (PfKH01_110029000), KH02 (PfKH02_110030000), IT (PfIT_110029200), but in none of the African isolates.

This collection of genomes is not intended to assess whether the distribution of genomic features are geographically structured. Nevertheless, a new gene on chromosome 11 was only found in non-African isolates within the panel and is absent from the 3D7 reference ( Figure 10). The coding sequencing of the gene is highly repetitive and contains many homopolymeric stretches. It has a weak Pfam hit (1e-5) to an MgtE intracellular N domain found at the N-terminus of magnesium transporters within eubacteria. I-TASSER predicts a potential transferase (C-score=-0.70; Estimated TM-score = 0.62±0.14 and Estimated RMSD = 11.3±4.5Å). However, it lacks both a signal peptide and transmembrane regions. The laboratory strains with American origin (Pf7G8 and PfHB3) have the gene. To shed further light on this, we determined the presence or absence of this gene in 2,500 samples from the Pf3K project. We found that it was present with a frequency of around 15% in South East Asia, compared to 6% in Malawi and less than 1% in other African samples ( Table 6). The gene is present as an intact copy in the two available samples from *P. reichenowi* ^39^, a closely related parasite of chimpanzees. In the *P. falciparum* genomes that contain the gene, it therefore appears to have at least one frameshift. However, these are highly likely to be sequencing errors because the repetitive nature of the coding sequence presented a problem for PacBio sequencing (and sequence correction). The complete absence of the whole sequence from most of the African isolates argues against the alternative explanation that a pseudogene is being differentially maintained in different populations. Its higher frequency in South East Asia and the Americas may be due to a founder effect, or relatively weak purifying selection to remove the gene in these populations, as has been seen for removal of genomic copy number variants ^40^.

**Table 6.** Frequency of new gene (PfIT_1100029200) in the Pf3k dataset, ordered by frequency.

Country	Total number of isolates	% Isolates with new gene
Laos	85	18.8
Myanmar	60	15.0
Cambodia	570	14.70
Thailand	148	14.20
Bangladesh	50	10.00
Vietnam	97	7.20
Malawi	369	5.40
Congo DR	113	2.70
The Gambia / Guinea	165	1.80
Ghana	617	1.10
Senegal	137	0.70
Mali	96	0.00

## Discussion

Using long read technology we have generated 15 new genome sequences of *P. falciparum* that reveal a highly conserved genome architecture with no large scale structural differences, and collectively provide an improved reference for this important parasite species.

A striking feature of *P. falciparum* chromosomes is that they each comprise a long interstitial region that is conserved across multiple species, flanked by large distal regions that contain polymorphic multigene families. Researchers have frequently referred to these regions as core and subtelomeric, respectively ^7, 41– 44^, yet it is surprising that there have been few attempts formally to demarcate their boundaries. The availability of high quality assemblies enables us to address this issue. The boundaries were previously assumed to be either the position of the last one-to-one orthologue between all *Plasmodium* genomes, or the first gene of one of the variable gene families, mostly *rifin* or *stevor* genes. Our new definition based on mapping is consistent with the hypothesis that the boundary of the core genome represents the last genes that are subject to consistent homologous recombination. This definition is only relevant at the population level because isolates that are the product of recent meioses will share extended regions of homology. Telomeric to the core regions, highly polymorphic variable gene families are found that show little sequence homology, some copy number variation but also considerable consistency in organisation such as the orientation of the most telomeric *var* gene. This suggests functional constraints to recombination also exist within the subtelomeric regions. The core region contains internal clusters of *var* genes that show positional conservation as well as a fairly consistent number and orientation.

The clone 3D7 does indeed appear to be an accurate reference for the species as a whole in terms of gene content and organisation. There are however some important exceptions. We identify a novel gene that has a higher frequency in Asia and a new member of the CLAG gene family. Given the disparate yet important functions of existing CLAG paralogues, establishing the role of this new member should be a priority.

## Data availability

The raw sequence data (Illumina and Pacific Bioscience reads) can be retrieved from the European Nucleotide Archive ( ERP009847); sample accession numbers (first Pacific Bioscience and then Illumina) Pf3D7: ERS782627 and ERS557779; IT: ERS480577 and ERS557780; Dd2: ERS639545 and ERS754081; HB3: ERS712858 and ERS754080; 7G8: ERS686280 and ERS754078; GB4: ERS686279 and ERS754079; Senegal: ERS637501 and ERS740935; Guinea: ERS739315 and ERS740940 Togo: ERS746008 and ERS747371; Mali: ERS755621 and ERS747373; Gabon: ERS739316 and ERS740939, Congo: ERS806988 and ERS1306147; Sudan: ERS746009 and ERS747372; Kenya: ERS525803 and ERS740937; Cambodia (KH01): ERS712859 and ERS747370; Cambodia (KH02): ERS686281 and ERS740936.

The genome sequences are available at EBI under the accession number ERP009847.

The annotation (embl) files of all isolates are available at: ftp://ftp.sanger.ac.uk/pub/project/pathogens/Plasmodium/falciparum/PF3K/. This page also has the OrthoMCL files used in the analysis. The genomes will be uploaded into PlasmoDB.

The IPA software is available on GitHub: https://github.com/ThomasDOtto/IPA. Version 1.0.1 was used for this work.

The software is also available on Zenodo: https://doi.org/10.5281/zenodo.806818 ^45^

License: GNU General Public License v3.0
