# Supplementary material for: Long read assemblies of geographically dispersed Plasmodium falciparum isolates reveal highly structured subtelomeres
Source: Wellcome Open Res. 2018 May 3;3:52. [Version 1] doi: 10.12688/wellcomeopenres.14571.1 (PMC5964635; doi:10.12688/wellcomeopenres.14571.1)
Supplement: Peer review history

# Peer review: Referee response for version 1

**DOI:** 10.21956/wellcomeopenres.15862.r33114

**Published:** 2018-05-23

**Authors:** Michael F. Duffy

**Affiliations:** 1 School of Biosciences, Bio21 Institute, University of Melbourne, Melbourne, Vic, Australia

Otto et al. provide very useful information about genome organisation in *P. falciparum*. Until now a single annotated complete *P. falciparum* assembly of the 3D7 clone and a partial assembly of the ItG clone have been widely used to map sequencing data. Genome sequence data from multiple other clones is available including smaller, assembled contigs that have been used to identify de novo assembled *P. falciparum* var genes from recombinogenic regions (e.g. Rask 2010 PLoS Comp Biol). However the genomic organisation of these contigs and their *var* genes remained unknown because assembly of the *P. falciparum* short reads is difficult in the low complexity intergenic sequence and in the recombinogenic regions containing var genes.

 Otto et al. combine Illumina short reads and PacBio long reads to assemble 15 *P. falciparum* genomes into large contigs. The experiments and analyses were performed to a high standard and the conclusions of the paper were justified. The PacBio sequences were prone to false indels in homopolymeric or AT repeats but if these were excluded the 3D7 PacBio assembly was faithful to the 3D7 reference genome giving confidence in the other assemblies.

 The 15 genomes shared a highly conserved core but as predicted from previous partial assemblies the subtelomeric regions and chromosome internal var gene clusters were divergent. The authors chose a region where synteny is lost for half the clones to partition the chromosomes into conserved core and divergent subtelomeric regions. The definition of these boundaries is very useful information that could aid attempts to define sequences associated with recombination or nucleation/maintenance of chromatin boundaries. Subtelomeric sequence context affects var gene activation and the assembly of the subtelomeres might also help future studies investigate this effect.

 A principal, convincing finding was that the new boundaries have reassigned several multi-gene families from the subtelomeric sites to the chromosome core. The comparison between multiple genomes was required for this formal proof of the subtelomeric boundary. However, it is unclear how this study has discriminated between the two proposed definitions (which require citations) of subtelomeric boundaries, i.e. “ *The boundaries were previously assumed to be either the position of the last one-to one orthologue between all Plasmodium genomes, or the first gene of one of the variable gene families, mostly rifin or stevor genes. Our new definition based on mapping is consistent with the hypothesis that the boundary of the core genome represents the last genes that are subject to consistent homologous recombination.*”. From fig 4 the chromosome 14 LH boundary appears to be at an exported protein coding gene in a conserved location, whether this gene is itself conserved or variant should be indicated, and the RH boundary is intergenic between an exported protein coding gene in a conserved location and either genes encoding exported proteins or rif genes. Clearer annotation in this figure of the genes “subject to consistent homologous recombination” might help interpretation.

 A gene is also reported that occurs in greater frequency in SE Asian than in African isolates. The gene has no known function and has assembled with a frameshift that the authors say may be due to problems with the assembly. This gene and a CLAG gene are the only novel genes reported so despite its lack of importance for the parasite it would be useful to Sanger sequence the gene to confirm whether it does indeed have a frameshift.

 The column 5 heading “var array” in table 5 should be defined in the table footnotes.

I have read this submission. I believe that I have an appropriate level of expertise to confirm that it is of an acceptable scientific standard.

# Peer review: Referee response for version 1

**DOI:** 10.21956/wellcomeopenres.15862.r33115

**Published:** 2018-05-18

**Authors:** Kirk W. Deitsch

**Affiliations:** 1 Department of Microbiology and Immunology, Weill Cornell Medical College, Cornell University, New York, NY, USA

The report by Otto and colleagues provides another excellent resource for the malaria community, in particular to researchers interested in chromosome structure and the variability found within the subtelomeric regions of the chromosomes. Using long-read technology, the authors have assembled complete subtelomeric regions from 15 parasite isolates, something that has not been possible using previously available genome sequencing and assembly methods. This enables researchers to understand more fully the variability in these regions and to better understand where the boundaries with the core genome exist. The authors have provided a clear description of their work and a solid assessment of the findings. Overall this is a nice addition to the literature.

 While the majority of the paper is easily understood, a few additional items could be addressed by the authors. 

1. The authors mentioned that they chose 5 long-maintained laboratory isolates and 10 recently cultured clinical isolates for analysis. Did they find any consistent differences in genome structure that might be indicative of long-term growth in culture? For example, it has long been speculated that loss of the genes kahrp, hrp2 and hrp3 (through truncation of the associated subtelomeric domains) provide parasites with a growth advantage in culture but should be selected against in the field. However, there are recent reports of loss of hrp2 and hrp3 in field isolates. Similarly, truncation of chromosome ends through “telomere healing”, which often leads to a loss of rep20 and var genes, was also speculated to be a consequence of long-term culture. Is there any evidence for more frequent loss of subtelomeres (rep20, vars, etc) in long-term cultured isolates? This might be discernable from the data in Figure 3B, however it is not clear which isolates have longer subtelomeric regions. 
2. It might be worth noting that in Figure 4, within the left end of chromosome 12, the core genome extends into a var gene. This might seem counterintuitive to some readers unless it is pointed out that this particular var gene is var2csa, a gene previously identified as “strain transcendent”. Its conservation is therefore expected. 
3. It is not clear what information in Figure 5 is biologically interesting and what is simply an assessment of the efficiency of the sequencing method. For example, a lack of telomere repeats indicates the sequencing did not extend all the way to the end of the chromosome (chromosomes are not thought to be stable in the absence of a telomere), while loss of Rep20 could be due to incomplete sequencing or the result of changes in the chromosome structure (eg the result of telomere healing). If there are no telomere repeats, it is not clear if a lack of Rep20 or var genes is biologically meaningful or simply the result of incomplete sequencing. This figure indicates that many of the assembled chromosomes lacked telomere repeats and are therefore incomplete. How will this affect the assessment of the size of each chromosome and the total number of var genes? The paper is written in a way that leads the authors to believe that the differences in the number of var genes shown in Table 2 is reflective of variability found in the field, however can this be said with confidence if many of the chromosome ends are not complete?

I have read this submission. I believe that I have an appropriate level of expertise to confirm that it is of an acceptable scientific standard.

# Peer review: Referee response for version 1

**DOI:** 10.21956/wellcomeopenres.15862.r33063

**Published:** 2018-05-09

**Authors:** David Serre

**Affiliations:** 1 Institute for Genome Sciences, University of Maryland School of Medicine, Baltimore, MD, USA

This is an straight-forward and interesting study describing the use of PacBio long reads to assemble the subtelomeric regions of several P. falciparum isolates. The data and analyses are sound and the conclusions interesting and well supported by the data. Overall no major comments. My main criticism is that some of the display items (e.g. T1, F1, T4, T5, F6) are not extremely informative and dilute the impact of the other figures. Could they be moved to supplemental information (not sure if this is supported by the journal)?

 Minor comments:

- On page 6: "Comparing all of the PacBio based assemblies..." I assume this include the reassembled 3D7 but this should be clearly stated...
- On page 7: "abrupt increase in recombination..." This is not clear to me whether this statement is entirely correct. Could the recombination rate be comparable to neighboring regions but, due to high sequence homology between repeated elements, lead to non-homologous recombination and therefore sequence divergence? Same comment with "recombinogenic" a bit later in the paragraph.
- On page 11: the way it is written, it seems like the novel CLAG gene is on different chromosomes depending on the clone analyzed. Is this correct? If yes, this should be further discussed.
- On page 13: please indicate in the text the number of non-African isolates carrying the new gene (X out of X).

I have read this submission. I believe that I have an appropriate level of expertise to confirm that it is of an acceptable scientific standard.
